# Hypoxia-inducible factor-1 α/platelet derived growth factor axis in HIV-associated pulmonary vascular remodeling

**DOI:** 10.1186/1465-9921-12-103

**Published:** 2011-08-05

**Authors:** Joel Mermis, Haihua Gu, Bing Xue, Fang Li, Ossama Tawfik, Shilpa Buch, Sonja Bartolome, Amy O'Brien-Ladner, Navneet K Dhillon

**Affiliations:** 1 Division of Pulmonary and Critical Care Medicine, Department of Medicine, University of Kansas Medical Center, 3901 Rainbow Blvd., Kansas City, KS 66160, USA; 2 Department of Molecular & Integrative Physiology, University of Kansas Medical Center, 3901 Rainbow Blvd., Kansas City, KS 66160, USA; 3 Pathology & Laboratory Medicine, University of Kansas Medical Center, 3901 Rainbow Blvd., Kansas City, KS 66160, USA; 4 Department of Pharmacology and Experimental Neuroscience, University of Nebraska Medical Center, 42nd and Emile, Omaha, NE 68198, USA; 5 Department of Medicine, 5323 Harry Hines Blvd., Dallas, TX 75390, USA; 6 The Affiliated Hospital of Ningxia Medical College, 804S. Shengli St., Yinchuan, Ningxia 750004, People's Republic of China

**Keywords:** lungs, endothelial cells, gp-120, oxidative stress

## Abstract

**Background:**

Human immunodeficiency virus (HIV) infected patients are at increased risk for the development of pulmonary arterial hypertension (PAH). Recent reports have demonstrated that HIV associated viral proteins induce reactive oxygen species (ROS) with resultant endothelial cell dysfunction and related vascular injury. In this study, we explored the impact of HIV protein induced oxidative stress on production of hypoxia inducible factor (HIF)-1α and platelet-derived growth factor (PDGF), critical mediators implicated in the pathogenesis of HIV-PAH.

**Methods:**

The lungs from 4-5 months old HIV-1 transgenic (Tg) rats were assessed for the presence of pulmonary vascular remodeling and HIF-1α/PDGF-BB expression in comparison with wild type controls. Human primary pulmonary arterial endothelial cells (HPAEC) were treated with HIV-associated proteins in the presence or absence of pretreatment with antioxidants, for 24 hrs followed by estimation of ROS levels and western blot analysis of HIF-1α or PDGF-BB.

**Results:**

HIV-Tg rats, a model with marked viral protein induced vascular oxidative stress in the absence of active HIV-1 replication demonstrated significant medial thickening of pulmonary vessels and increased right ventricular mass compared to wild-type controls, with increased expression of HIF-1α and PDGF-BB in HIV-Tg rats. The up-regulation of both HIF-1α and PDGF-B chain mRNA in each HIV-Tg rat was directly correlated with an increase in right ventricular/left ventricular+septum ratio. Supporting our *in-viv*o findings, HPAECs treated with HIV-proteins: Tat and gp120, demonstrated increased ROS and parallel increase of PDGF-BB expression with the maximum induction observed on treatment with R5 type gp-120_CM_. Pre-treatment of endothelial cells with antioxidants or transfection of cells with HIF-1α small interfering RNA resulted in abrogation of gp-120_CM _mediated induction of PDGF-BB, therefore, confirming that ROS generation and activation of HIF-1α plays critical role in gp120 mediated up-regulation of PDGF-BB.

**Conclusion:**

In summary, these findings indicate that viral protein induced oxidative stress results in HIF-1α dependent up-regulation of PDGF-BB and suggests the possible involvement of this pathway in the development of HIV-PAH.

## Introduction

The advent of antiretroviral therapy (ART) has clearly led to improved survival among HIV-1 infected individuals, yet this advancement has resulted in the unexpected consequence of virus-associated noninfectious complications such as HIV-related pulmonary arterial hypertension (HIV-PAH) [1,2]. Despite adherence with ART, development of HIV-PAH serves as an independent predictor of death in patients with HIV-infection [3]. A precise characterization of the pathogenesis of HIV-PAH has so far proven elusive. As there is little evidence for direct viral infection within the pulmonary vascular bed [4-7], popular hypothesis is that secretary HIV-1 viral proteins in circulation are capable of inducing vascular oxidative stress and direct endothelial cell dysfunction and smooth muscle cell proliferation critical to the development of HIV-related arteriopathy [8,9]. Further, evidence is accumulating which suggests that the HIV-1 infection of monocyte/macrophages and lymphocytes stimulates increased production of pro-inflammatory markers and/or growth factors. implicated in the pathogenesis of HIV-PAH such as platelet derived growth factor (PDGF)-BB [10-16]. These soluble mediators can then initiate endothelial injury followed by smooth muscle cell proliferation and migration [2,17,18].

Previous studies provide evidence for the possible involvement of PDGF in the pathogenesis of pulmonary vascular remodeling in animal models [19,20] and in lung biopsies from patients with PPH or with HIV-PAH [12]. Furthermore, a non-specific inhibitor of PDGF signaling, imatinib, has demonstrated the ability to diminish vascular remodeling in animal studies and to mitigate clinical decline in human PAH trials [21-24]. Our previous work demonstrates an over-expression of PDGF *in-vitro *in HIV-infected macrophages [25] and *in-vivo *in Simian HIV-infected macaques [16]. Our recent work supports an HIV-protein mediated up-regulation of PDGF-BB in un-infectable vascular cell types such as human primary pulmonary arterial endothelial and smooth muscle cells [26]. However, the mechanism(s) by which HIV infection or viral protein(s) binding induces PDGF expression and the role of this potent mitogen in the setting of HIV-associated pulmonary arteriopathy has not been well characterized. HIV associated viral proteins including Tat and gp-120 have demonstrated the ability to trigger the generation of reactive oxygen species (ROS) [27,28]. As oxidative stress stabilizes hypoxia inducible factor (HIF)-1α, a transcription factor critical for regulation of important proliferative and vaso-active mediators [29-31], we hypothesize that viral protein generated reactive oxygen species (ROS) induce HIF-1α accumulation, with a resultant enhanced transcription of PDGF-B chain.

Thus, given the need for clarification of the mechanisms responsible for HIV-related pulmonary vascular remodeling, we, in the present study, first utilized the non-infectious NL4-3Δ*gag/pol *HIV-1 transgenic (HIV-Tg) rat model [32,33] to explore the direct role of viral proteins in the development of pulmonary vascular remodeling. This HIV-Tg rat model [34], develops many clinical multisystem manifestations similar to those found in AIDS patients and most importantly, has earlier been demonstrated to be under significant oxidative stress. Furthermore, given that the pulmonary artery endothelial dysfunction plays a key role in the initiation and progression of PAH [35-37], utilizing the primary pulmonary endothelial cell-culture system we next delineated the importance of oxidative stress and HIF-1α activation in viral protein mediated up-regulation of PDGF-BB.

## Methods

### HIV-1 transgenic and wild type rats

HIV-1 transgenic (Tg) Sprague Dawley (SD) and SD wild type (WT) rats were purchased from Harlan (Indianapolis, Indiana). Young 4-5 months old Tg rats (*n *= 6) and age matched SD wild type rats (*n *= 6) were used for analysis. The HIV-1 Tg rat contains a *gag-pol *deleted NL4-3 provirus and expresses HIV viral RNA and proteins in various tissues including lymphocytes and monocytes. The animals were euthanized with inhalation of 2.5-3% isofluorane gas, followed by transcardial saline perfusion. Following euthanasia, one half of the lung was post-fixed for histological examination, while the other half was snap frozen for RNA analysis. The animal care at the Kansas University Medical Center was in strict accordance with the National Institutes of Health (NIH) Guide for the Care and Use of Laboratory Animals.

### Right Ventricular Mass Evaluation

Hearts were removed from the euthanized animals. After the removal of atria, the wall of the right ventricle (RV) was separated from the left ventricle (LV) and septum (LV+S) according to the established method [38]. Wet weights of both RV & LV+S were quantified, normalized to the total body weight and used to calculate the RV/LV+S ratio.

### Histology and immuno-histochemical analysis of pulmonary arteries

Excised lungs were immersed in 4% paraformaldehyde overnight followed by 70% ethanol and then used for paraffin embedding. Paraffin sections of 5 μm thickness were used for Hematoxylin & Eosin (H&E) or Verhoeff von Gieson (VVG) staining. The digital scans of whole section from each animal were generated with a ScanScope scanner and then visualized and analyzed using Aperio image view software. VVG-stained sections from each animal were evaluated for medial wall thickness of pulmonary arteries (50-250 μm diameter) in blinded manner. Wall thickness and outer diameter of approximately 25 muscularized arteries were measured in each section at two perpendicular points and then averaged. The percentage medial wall thickness was then calculated as described before [39]. Immunohistochemistry staining of paraffin-embedded lung sections was performed as previously described [16] with primary antibodies including α-SMA, factor VIII, from Dako Corporation (Carpentaria, CA, USA), HIF-1α, from Santa Cruz Biotechnology, Inc. (Santa Cruz, CA) and PDGF-BB, from Abcam, Inc. (Cambridge, MA).

### Cell culture and treatments

Human primary pulmonary microvascular endothelial cells (HPMVEC) were purchased from ScienCell research laboratories (Carlsbad, CA) and grown in endothelial cell basal media containing 2% fetal bovine serum (FBS), 1 μg/ml hydrocortisone, 10 ng/ml human epidermal growth factor, 3 ng/ml basic fibroblast growth factor, 10 μg/ml heparin, and gentamycin/amphotericin. Cells were treated with viral proteins: Tat 1-72 (1 μM, University of Kentucky), gp-120_CM _or gp-120_LAV _(100 ng/ml, Protein Sciences Corporation, Meriden, CT) for 24 hrs or 1 hr followed by western blot analysis and ROS quantification, respectively. Tat or gp120 stock solution was heat-inactivated by boiling for 30 min. For treatment with CCR5 neutralizing antibody or IgG isotype control (10 μg/ml, R&D systems) or; with antioxidant cocktail (0.2 mM ascorbate, 0.5 mM glutathione, and 3.5 μM α-tocopherol), cells were pre-treated with inhibitors for 30 min followed by treatment with gp-120 _CM._

### Quantification of cellular oxidative stress using dichlorofluorescein (DCF) assay

Pulmonary endothelial cells were treated with 5-(and -6)-carboxy-2', 7'-dichlorodihydroflourescein diacetate (DCFH-DA) (Molecular Probes, Inc.) for 30 min followed by treatment with viral protein(s) for 1 hr. In the presence of H_2_O_2_, DCFH is oxidized to a fluorescent DCF within the cytoplasm which was read by fluorescent plate reader at an excitation of 485 nm with an emission of 530 nm [40].

### Transfection of pulmonary endothelial cells with small interfering (si) RNA

The silencer select pre-designed and validated siRNA duplexes targeting HIF-1α were obtained from Applied Biosystems (Carlsbad, CA). Cells were also transfected with silencer select negative control siRNA for comparison. HPMVECs were transfected with 10 nM siRNA using Hiperfect transfection reagent (Qiagen, Valencia, CA) as instructed by the manufacturer. The transfected cells were then treated with or without cocaine and/or Tat for 24 hrs followed by protein extraction for western blot analysis.

### Real-Time RT-PCR analysis

We used Real-Time RT-PCR to analyze RNA extracted from frozen lungs of HIV-1 Tg rats and WT controls obtained after non-fixative perfusion. Quantitative analysis of HIF-1a, PDGF and ET-1 mRNA in Tg and WT rats was done using primers from SA Biosciences (Frederick, MD) by Real-Time RT-PCR using the SYBR Green detection method. Total RNA was isolated from frozen lung tissues by lysis in Trizol and was then converted into first strand cDNA to be used for real-time PCR. Detection was performed with an ABI Prism 7700 sequence detector. The average Ct value of the house-keeping gene, HPRT, was subtracted from that of target gene to give changes in Ct (dCt). The fold-change in gene expression (differences in dCt, or ddCt) was then determined as log2 relative units.

### Western Blot Analysis

Frozen rat lung tissues or endothelial cells were lysed in lysing buffer (Sigma, St. Louis, MO) containing protease inhibitors. Protein estimation in these samples was measured using the micro-BCA method protein assay kit (Pierce Chemical Co., Rockford, IL). Western blot analyses were performed using primary antibodies against HIF-1α (Santa Cruz), PDGF-BB (Santa Cruz), and β-actin (Sigma). The secondary antibodies used were horseradish peroxidase-conjugated anti-mouse or anti-rabbit (1:5000, Pierce Chemical Co) and detection was performed using the enhanced chemiluminescence system (Pierce Chemical Co.). The NIH imageJ software was used for densitometric analysis of immunoblots.

### Statistical Analysis

Statistical analysis was performed using two-way analysis of variance with a post-hoc Student t- test or non-parametric Wilcoxon Rank-Sum test as appropriate. To test for association of RV/LV+septum ratio with other mediators, the non-parametric Spearman's rank-sum correlation coefficient was used and coefficient of determination (R^2^) was calculated. Exact two-sided p-values were calculated for all the analysis, using SAS 9.1 software (SAS Institute, Inc., Cary, NC, USA). A type I error rate of 5% was used for determining statistical significance.

## Results

### Pulmonary vascular remodeling in HIV-Tg rats

**Figure 1:**
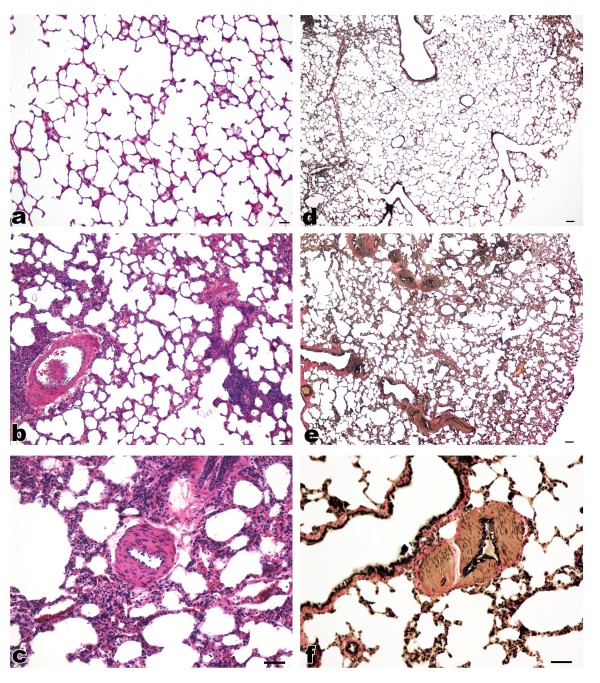
**Histological evidence of pulmonary vascular remodeling in HIV-Tg rats**. Representative images of H& E (**a, b, c**) and VVG (**d, e, f**) stained sections from HIV-Tg (b, c, e, f) and WT control (a, d) rats. H&E photomicrographs were captured at 10× (a, b) and at 20× (c) magnification whereas VVG images were captured at 4× (d, e) and at 20× (f) original magnification (scale bar: 100 μm). Each representative image is from different animal.

**Figure 2:**
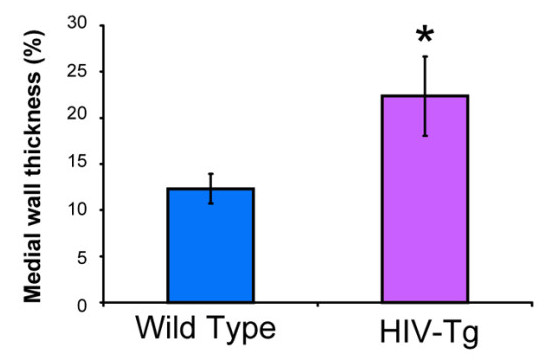
**Increase in medial wall thickness of pulmonary arteries in HIV-Tg rats compared with WT-rats**. VVG-stained sections from each animal were evaluated for medial wall thickness of pulmonary arteries with diameter size ranging from 50 μm-250 μm. P < = 0.001, HIV-Tg rats vs. WT controls.

Reports suggesting respiratory difficulty in HIV-Tg rats [34] led us to utilize this model in looking for evidence of pulmonary arteriopathy associated with HIV-related proteins. As clinical manifestations of AIDS in HIV-Tg rats begins as early as 5 months [34], we compared 4-5 months old Tg with age-matched WT-control rats (*n *= 6 in each group). Analysis of both, H&E and VVG staining, of paraffin embedded lung sections from 5 months old HIV-Tg rats demonstrated moderate to severe vascular remodeling. Representative images of H&E and VVG staining from each group are shown in Figure 1A. There was a significant increase in the thickness of the medial wall of muscular arteries in HIV-Tg rats (Figure 1b, c, e, f) compared to normal vessels in wild-type control (Figure 1a, d). Further, the presence of a smooth muscle layer was observed in many of the normally non-muscular distal arteries of HIV-Tg rats. The VVG-stained sections from both the groups revealed a well defined internal elastic lamina (black stain) in WT-control rats whereas elastic lamina was found to be disrupted in HIV-Tg rats (Figure 1). As shown in Figure 2, percentage of medial wall thickness of pulmonary arteries with outer diameter ranging between 50-200 μm was observed to be significantly high in HIV-Tg rats as compared to WT controls (p < 0.001).

### Characterization of pulmonary vascular lesions in HIV-Tg rats

**Figure 3:**
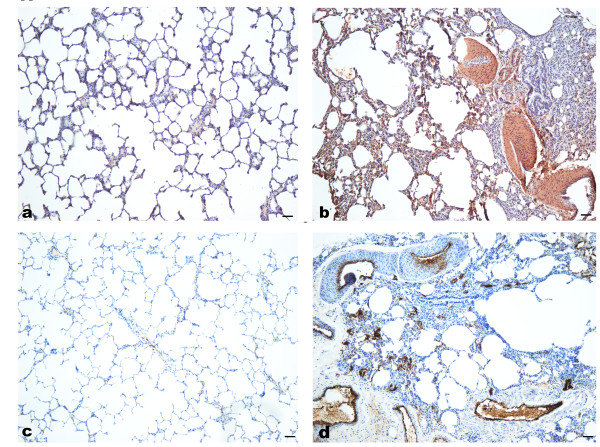
**Presence of medial wall thickness in the pulmonary arteries of HIV-Tg rats**. Immuno-histochemistry of paraffin embedded lung sections with anti- α smooth muscle actin (brown) (a-b) and factor VIII (brown) (c-d) indicated abnormal vascular lesions with significant medial wall thickening in the lung sections from HIV-Tg rats (b, d) compared with WT-controls (a, c). Representative images were captured at 10× original magnification (scale bar: 100 μm).

In order to characterize the cellular composition of pulmonary vascular lesions in HIV-Tg rats; the lung sections were stained for α-SMA and Factor VIII. As shown in the representative lung sections from each group in Figure 3, we confirmed the presence of vascular remodeling with medial wall thickness in HIV-Tg rats while normal blood vessels were observed in WT control rats. Marked increase in the medial wall thickness of muscular arteries was observed due to increased proliferation of smooth muscle cells (SMC) in the HIV-Tg group (Figure 3b) compared to WT controls (Figure 3a). Endothelial monolayer was generally damaged with signs of increased expression of factor 8 (or vWF) in both thickened and non-muscular vessels (Figure 3d).

### Right ventricular hypertrophy (RVH) in HIV-Tg rats

**Figure 4:**
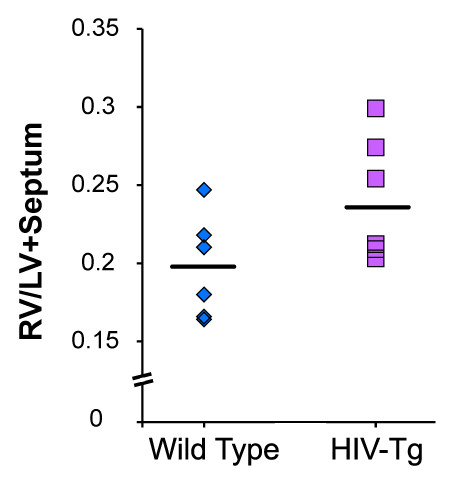
**Right ventricular hypertrophy in HIV-Tg rats (*n *= 6) compared with age-matched WT rats (*n *= 6)**. The ratio of the wet weight of RV wall and of the LV wall with septum of heart (RV/LV+septum) was measured. P = 0.06, HIV-Tg rats vs. WT controls.

The HIV-Tg rats exhibited an increase in the ratio of wet weight of the right ventricle (RV) to the sum of the wet weights of the left ventricle and interventricular septum (LV+S), compared to that found in the control group (Figure 4). This increase in the RV/LV+S ratio suggests a disproportionate growth of the right ventricle compared to the left, thereby indicating early RV hypertrophy in these HIV-Tg rats.

### Increased expression of HIF-1a and PDGF-BB in HIV-Tg rats

**Figure 5:**
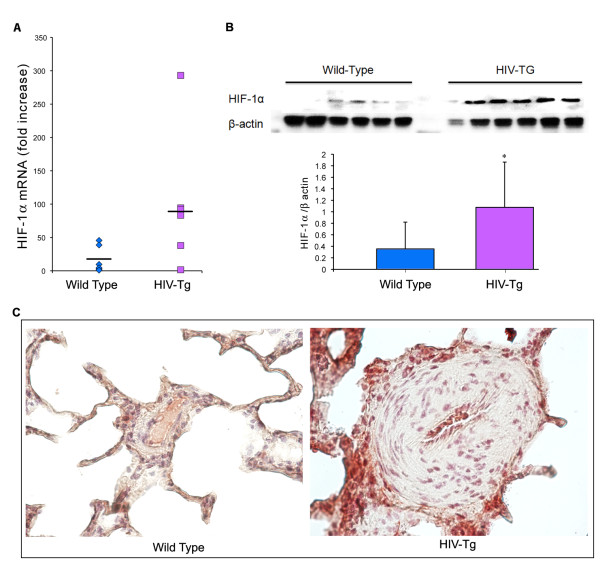
**Increased expression of HIF-1α in HIV-Tg rats compared to wild type controls**. **A) **Real-Time RT-PCR analysis of total mRNA and **B**) western blot analysis of total protein, extracted from lungs of HIV-Tg rats and age matched wild-type SD rats. Histogram below western blot image represents the average densitometric ratio of 135 kDa, HIF-1α to β-actin in wild-type and HIV-Tg rats. Statistical significance was calculated using a two-tail, independent, t-test. (* p < = 0.05) **C) **Immuno-histochemistry of paraffin embedded lung sections with anti-HIF-1α. Representative photomicrographs of immunostaining from wild-type and HIV-Tg group are shown. Original magnification: 60×.

Having determined, through observation of right ventricular changes, the degree of pulmonary arteriopathy in HIV-Tg rats, we next compared the level of HIF-1α expression in the lungs of these rats to that found in controls. Although RNA analysis of lung extracts suggested an insignificant increase in the expression of HIF-1α (p = 0.078) (Figure 5A), western blot analysis demonstrated significant (p < 0.05) increase in HIF-1α protein, thus confirming the increase in expression of HIF-1α in HIV-Tg rats as compared to WT controls (Figure 5B). This increase in the expression of HIF-1α was further confirmed by immunohistochemical analysis on the lung sections from HIV-Tg and WT controls. As shown in Figure 5C, the lung parenchyma, along with endothelial cells lining the vessels demonstrating medial thickness, had strong expression of HIF-1α in the lung sections from HIV-Tg rats. Enhanced expression was also observed in mononuclear cells around the thickened vessels. Smooth muscle cells in these arteries, however, did not demonstrate a significant increase in HIF-1α compared to those from controls.

**Figure 6:**
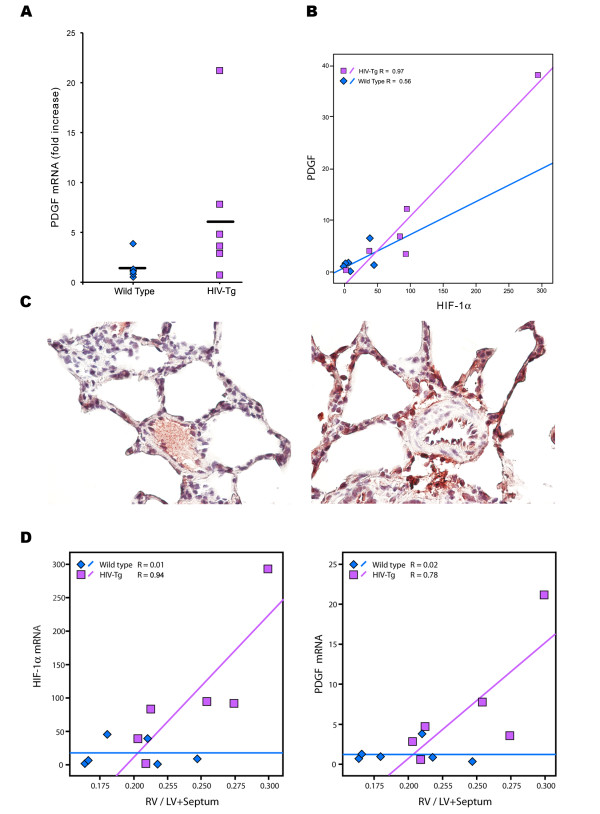
**Increased expression of PDGF-B chain in HIV-Tg rats compared to wild type controls**. **A) **Real-Time RT-PCR analysis of total mRNA in the lungs of HIV-Tg rats and age matched wild-type SD rats. **B) **Correlation of PDGF-B chain mRNA with the expression of HIF-1α in HIV-Tg rats. **C) **Immuno-histochemistry for PDGF-BB on the paraffin embedded lung sections from HIV-Tg and WT rats. Original magnification: 60×. **D) **Correlation of RV/LV+S ratio with the expression of HIF-1α and PDGF-BB in HIV-Tg rats. Correlation was calculated using the non-parametric Spearman's rank correlation coefficient.

We next evaluated the expression of pro-proliferative factor, PDGF-BB that is suggestive to be regulated in a HIF-dependent manner [29-31]. As shown in Figure 6A, real-time RT-PCR analysis of total mRNA extracted from lung homogenates suggested an increase in the expression of PDGF-B chain mRNA in HIV-Tg rats compared to the control rats with normal vasculature. Interestingly, this increased expression of PDGF-B chain in the HIV-Tg group was associated positively with the increase in expression of HIF-1α (p = 0.002, R = 0.97) (Figure 6B). Furthermore, immunohistochemical analysis suggested enhanced expression of PDGF-BB in endothelial cells and in mononuclear infiltrated cells around thickened vessels (Figure 6C) from HIV-Tg rats similar to HIF-1α staining (Figure 5C). Additionally, the increased levels of HIF-1α (p = 0.009, R = 0.94) and PDGF-B chain (p = 0.036, R = 0.78) strongly correlated linearly with the increased RV/LV+ septum ratio in HIV-Tg rats (Figure 6D). No notable trends were found within the wild-type group.

### Increased expression of PDGF-BB in HIV-protein(s) treated pulmonary microvascular endothelial cells

**Figure 7:**
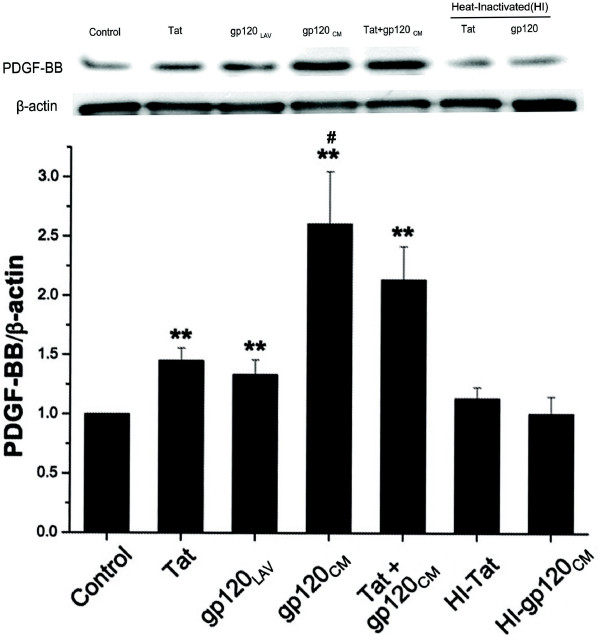
**Increased expression of PDGF-BB in pulmonary endothelial cells on treatment with HIV-proteins**. Representative western blot showing PDGF-BB expression in cellular extracts from Tat (25 ng/ml), gp-120_LAV _(100 ng/ml), gp-120_CM _(100 ng/ml), heat-inactivated (HI) Tat or HI-gp-120 treated human pulmonary microvascular endothelial cells. The blots were re-probed with human β-actin antibodies. Histogram represents the average densitometric ratio of PDGF-BB to β-actin of three independent experiments. Statistical significance was calculated using a two-tail, independent t-test. (** p ≤ 0.01 vs. control, #p ≤ 0.05 vs. Tat or gp-120 treatment).

Since we observed increased expression of HIF-1α and PDGF-BB in the lungs from HIV-Tg rats including endothelial cells lining the pulmonary arterial vessels, we next sought to delineate if HIF-dependent mechanism is involved in the viral protein mediated up regulation of PDGF-BB in the pulmonary endothelium. Two major HIV-proteins: Tat and gp-120 are known to be actively secreted by infected cells and has been detected in the serum of HIV-infected patients [41-43]. Furthermore, given that both Tat and gp-120 were found to express in the lung homogenates of HIV-Tg rats (data not shown), we first treated HPMVECs with these viral proteins over a period of 24 hrs and assessed for the expression of PDGF-BB by western blot analysis. As shown in Figure 7, treatments with Tat, gp-120_LAV _(from X4-type virus) or gp-120_CM _(from R5-type virus) resulted in significant increase of PDGF-BB protein expression compared to untreated control. However, when cells were subjected to treatment with the same concentration of heat inactivated Tat or gp-120, no induction in the PDGF-BB expression was observed. Additionally, the maximum increase that was observed on treatment with R5-type gp-120_CM _was also significantly more when compared with the PDGF-BB induction obtained on Tat or X4-type gp-120_LAV _treatment.

### Reactive oxygen species are involved in HIV-protein mediated PDGF-BB induction

**Figure 8:**
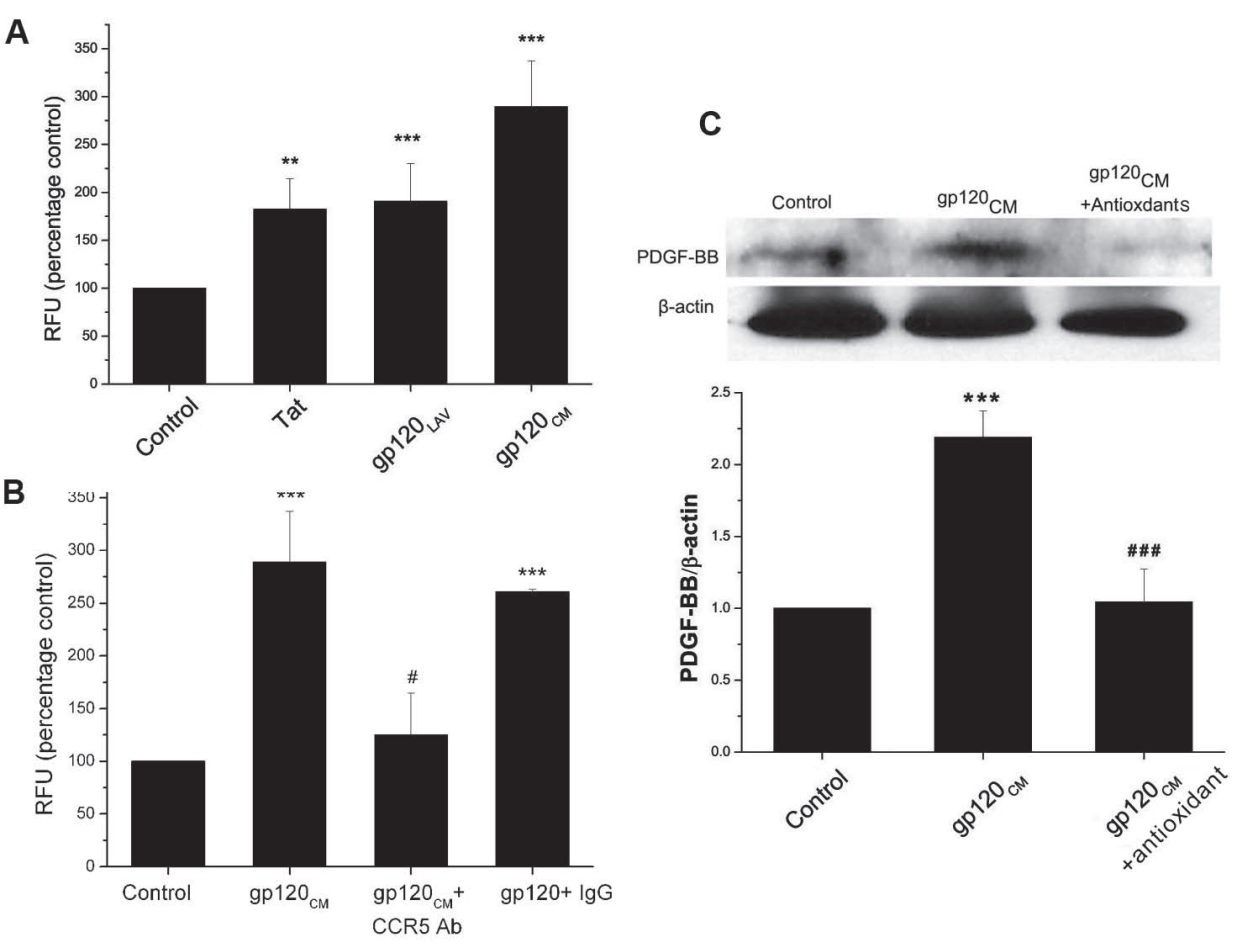
**Involvement of oxidative stress in gp-120 mediated PDGF-BB induction in pulmonary endothelial cells**. **A) **Enhanced oxidative stress in pulmonary endothelial cells on Tat and gp120 treatment. Human pulmonary microvascular endothelial cells (HPMVECs) were incubated with carboxy-H2-DCF-DA followed by Tat (25 ng/ml) or gp-120 (100 ng/ml) treatment for 60 min, and assessed for oxidative stress (Mean ± SD., **P ≤ 0.01, ***P < 0.001 vs. control). **B) **Effect of CCR5 neutralizing antibody on gp-120_CM _(100 ng/ml) mediated oxidative stress in HPMVECs. Cells were pretreated with CCR5 antibody (10 μg/ml) or equal amount of IgG isotype control for 30 min, followed by DCF assay (Mean ± SD., ***P < 0.001 treatment versus control; #P < 0.05 vs. gp120_CM _treatment). **C) **Gp-120_CM _mediated PDGF-BB expression in the presence of antioxidant cocktail. HPMVECs were pretreated with antioxidant cocktail for 30 min followed by incubation with gp-120_CM _(100 ng/ml) for 24 hours. Cells were then used for protein extraction followed by sequential immunobloting with antibodies specifically directed to the PDGF-BB and β-actin. Representative western blot images (upper panel) are shown with histograms (lower panel) showing the average densitometric analysis of the PDGF-BB band normalized to corresponding β-actin band from three independent experiments (*** P < = 0.001 versus control; ###P < = 0.001 versus gp120_CM _treatment).

Since both Tat and gp-120 [27,28,44] are known to induce oxidative stress, we next evaluated the levels of cytoplasmic ROS in Tat or gp-120 treated HPMVECs by DCF assay. Our findings demonstrated that the treatment of cells with Tat, gp-120_LAV _or gp-120_CM _results in significant increase in the production of ROS when compared to controls (Figure 8A). Similar to the PDGF-BB expression the maximum oxidative burst was observed on treatment with R5 type gp-120_CM_. Based on these findings we next focused on elucidating the mechanism(s) involved in the gp-120_CM _mediated up-regulation of PDGF-BB in pulmonary endothelial cells. We first investigated if chemokine receptor CCR5 is specifically involved in gp-120_CM _mediated generation of ROS by use of CCR5 neutralizing antibody. As illustrated in Figure 8B, pretreatment of HPMVECs with CCR5 antibody for 30 min, prevented the ROS production on gp-120 _CM _treatment whereas the pretreatment with isotype matched control antibody control had no affect. Furthermore, to examine if this enhanced levels of ROS are involved in PDGF-BB increase associated with gp-120_CM _treatment of pulmonary endothelial cells, cells were pretreated with antioxidant cocktail for 30 min. followed by 24 h treatment with gp-120_CM_. As shown in Figure 8C, western blot analysis of the total cell extract demonstrated the ability of antioxidants to prevent the gp-120 _CM _mediated increase in the PDGF-BB expression. Taken together these data suggest the role of oxidative burst in R5-type gp-120 mediated up-regulation of PDGF-BB in pulmonary endothelial cells.

### ROS dependent stimulation of HIF-1α is necessary for HIV-protein mediated PDGF-BB induction

**Figure 9:**
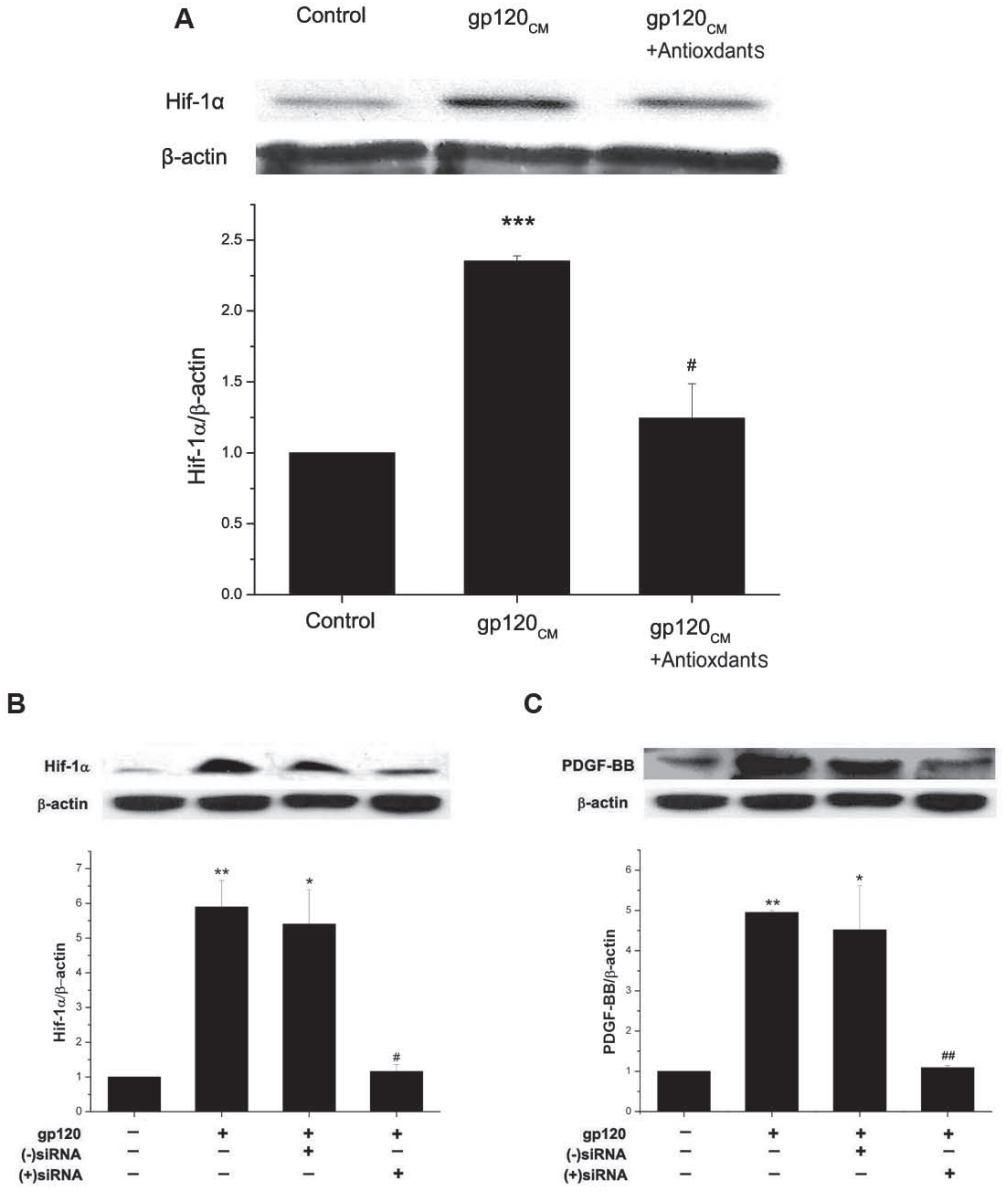
**Oxidative stress dependent HIF-1α expression is involved in gp-120_CM _mediated PDGF-BB induction**. **A) **Western blot analysis of HIF-1α expression in human pulmonary microvascular endothelial cells (HPMVECs) pretreated with or without antioxidant cocktail for 30 min followed by incubation with gp-120_CM _(100 ng/ml) for 24 hours. **B) **Evaluation of HIF-1α knockdown by western blot analysis of whole cell lysates from HPMVECs transfected with siRNA specific to HIF-1α (10nM) or with negative control siRNA in presence of gp120_CM _treatment. (**C) **Knock down of HIF-1α resulted in inhibition of gp120_CM_-mediated induction of PDGF-BB expression in HPMVECs. Blots are representative of three independent experiments with histogram (lower panel) showing the average densitometric analysis normalized to β-actin. All values are mean ± SD. *P < = 0.01,**P < = 0.001 treatment versus control; #P < = 0.01, ##P < = 0.001 treatment versus gp120_CM _treated untransfected cells.

It is well known that most of the pathological effects of ROS in various oxidative stress associated disorders are mediated by activation and stabilization of HIF-1α [45]. We therefore next investigated if ROS mediated activation of HIF-1α on gp-120_CM _treatment is important for increased expression of PDGF-BB in gp-120 treated HPMVECs. We first examined whether gp-120_CM _treatment of HPMVECs could result in increased levels of HIF-1α protein. As shown in Figure 9A, western blot analysis of gp-120_CM _treated cellular extracts demonstrated increased levels of HIF-1α as compared to untreated controls. Furthermore, this gp-120_CM _mediated induction of HIF-1α expression was inhibited on pre-treatment of HPMVECs with an antioxidant cocktail (Figure 9A), thus confirming the ROS mediated augmentation of HIF-1α expression on R5 gp-120 treatment of endothelial cells.

Next to determine the involvement of HIF-1α in gp-120 mediated regulation of PDGF-BB expression, we used HIF-1α specific siRNA knock down experiments. First we optimized that the transfection of HPMVECs with 10nM HIF-1α siRNA was efficient in decreasing around 80% of gp-120_CM _induced HIF-1α expression when compared to cells transfected with non-specific siRNA control (Figure 9B). Furthermore, the HIF-1α siRNA transfected cells showed significant decrease in the expression of PDGF-BB in the presence of gp-120_CM _when compared with untransfected or non-specfic siRNA transfected gp-120_CM _treated cells (Figure 9C) thus underscoring the role of HIF-1α activation in the gp-120_CM _mediated PDGF-BB expression in pulmonary endothelial cells.

## Discussion

In this study, we offer histological and physiologic evidence of pulmonary vascular remodeling with significant thickening of medial layer of the arteries, elevated RV mass in the non-infectious rat model of HIV-1. Pulmonary arteriopathy exhibited by the HIV-Tg rats was manifested primarily by smooth muscle proliferation within the medial wall, endothelial disruption with little indication of endothelial cell proliferation but absence of classic plexiform lesions. Although RV hypertrophy in the HIV-Tg rats is suggestive of concomitant right heart pressure overload, the presence of pulmonary arteriopathy alone does not necessarily predict pulmonary hypertension. Furthermore, in humans only a fraction of individuals with HIV develop PAH, suggesting that the etiology of HIV-PAH is multi-factorial and complex where multiple insults such as HIV infection, drugs of abuse, and genetic predilection may be necessary to induce clinical disease. Therefore, one could hypothesize that the viral protein(s) provides the first 'hit' and second 'hit' such as administration of stimulants may lead to more severe pathology in these HIV-Tg rats.

Inflammation is considered to play an important role in HIV- associated pulmonary vascular remodeling with accumulation of macrophages and T lymphocytes found in the vicinity of pulmonary vessels of pulmonary hypertension patients [46,47]. Consistent with these findings we also observed infiltration of mononuclear cells near or around the thickened vessels with mild interstitial pneumonitis as described before in this model [34]. HIV-1 infection is known to stimulate monocyte/macrophages and lymphocytes to secrete elevated levels of cytokines, growth factors and viral proteins such as Nef, Tat and gp-120, [10-16] that can then initiate endothelial injury, SMC proliferation and migration, leading to the development of HIV-PAH [8-10,18,26,48]. It is plausible that the medial wall thickening, an important determinant of pulmonary vascular resistance, discovered in HIV-Tg rat model is the result of integrated effects of various HIV proteins and the related inflammatory mediators including PDGF-BB.

Examination of the HIV-1 Tg rat lungs revealed increased staining of PDGF-BB in macrophages around hypertrophied vessels and in endothelial cells. Earlier studies suggest induction of PDGF-BB by endothelial cells [49] but not by SMCs [50] in response to hypoxia. Furthermore, the vasculature and lungs of this HIV-Tg rat model has earlier been demonstrated to be under significant oxidative stress [32,33]. Along the lines, we observed enhanced expression of HIF-1α, a crucial transcription factor responsible for sensing and responding to oxidant stress and hypoxic conditions [51], suggesting, in part, the involvement of ROS/HIF-1α pathway in the over expression of PDGF-BB. HIF-1α controls a large program of genes critical to the development of pulmonary arterial hypertension [29,31,52,53]. Interestingly, the expression of HIF-1α and PDGF was not only elevated and positively associated with each other in the lungs of the HIV-1 Tg rats, but the quantity of each was directly related, in a linear fashion, to the degree of increase in the right ventricular hypertrophy (RV/LV+septum ratio).

While these correlations in the HIV-1 transgenic model are consistent with our hypothesis, our *in-vitro *work in pulmonary endothelial cells validates that viral protein mediated oxidative stress/HIF-1α pathway results in induction of PDGF-BB. The injury to the endothelium, an initiating event in PAH [54] is known to be associated with the induction of oxidative stress [44]. HIV-associated proteins, Tat and gp-120, as confirmed by our findings and others, demonstrate the ability to invoke oxidative stress mediated endothelial dysfunction [27,28,44,55]. In addition, results demonstrating enhanced levels of HIF-1α in viral protein treated pulmonary endothelial cells, are in concert with the previous findings supporting the activation and accumulation of HIF-1α by HIV-1 through the production of ROS [56]. PDGF-BB, known to be involved in hypoxia-induced vascular remodeling, [30,57,58]) has been suggested to be up-regulated in a HIF-dependent manner, but the mechanism by which HIF-1α and PDGF levels are elevated during vascular remodeling associated with PAH, are still not completely understood. A putative HIF-response element on PDGF-gene has been identified [59] but the studies demonstrating the direct involvement of HIF-1α in the regulation of PDGF expression are lacking. Here, we provide evidence validating the significance of HIF-1α in the pathogenesis of HIV-associated vascular dysfunction, and report the novel finding that its response to viral protein generated oxidative stress is to augment PDGF expression in the pulmonary endothelium. To our knowledge, this is the first report validating that HIV-1 viral proteins through the activation of HIF-1α induce PDGF expression.

The HIV-1 virus is unable to actively infect endothelial cells due to the absence of necessary CD4 receptors. However, viral proteins have been demonstrated to act on endothelial cells through direct binding to their CCR5 (R5) or CXCR4 (X4) co-receptors [60]. This is corroborated with our findings showing mitigation of gp-120 _CM _response to increase PDGF-BB expression in the presence of CCR5 neutralizing antibody. Maximum PDGF-BB expression and ROS production was seen on treatment with R5-type gp-120 that is expected to be secreted in abundance by infiltrated HIV-infected CCR5+ T cells [61] and macrophages seen around the pulmonary vascular lesions associated with PAH [62]. In addition, studies on co-receptor usage of HIV have shown that virus utilizing CCR5 as a co-receptor is the predominant type of virus found in HIV-infected individuals [63]. Furthermore, R5gp120 has been reported earlier to induce the expression of cell-cycle and cell proliferation related genes more strongly than X4 gp-120 in peripheral blood mononuclear cells [64] and this differential potency of gp-120 effect may be present in pulmonary endothelial cells as well.

## Conclusion

In summary, we demonstrate that the influence of HIV-1 proteins alone, without viral infection, is associated with pulmonary arteriopathy including accumulation of HIF-1α and PDGF as observed in the HIV-1 Tg rats. Furthermore, our *in-vitro *findings confirm that HIV-1 viral protein mediated generation of oxidative stress and resultant activation of HIF-1α leads to subsequent induction of PDGF expression in pulmonary endothelial cells. Consistent with a possible role of PDGF in the development of idiopathic PAH, the correlation of this mediator with RVH, does suggest this pathway may be one of the many insults involved in the development of HIV-related pulmonary arteriopathy and potentially HIV-PAH.

## Competing interests

The authors declare that they have no competing interests.

## Authors' contributions

All authors have read and approved the manuscript. JM contributed in writing manuscript, quantitated medial wall thickness and participated in data analysis; HG performed all the cell-culture experiments; XB performed immuno-histochemistry and western blots on rat lungs; FL harvested lung tissues, extracted RNA and performed real-time RT-PCR experiments; OT reviewed the H&E and immunohistochemical stained sections, SJB and SB contributed in critiquing the manuscript; AL participated in interpretation of the data and writing the manuscript; NKD designed the study and supervised overall experimental plans, analyzed and interpreted the data, and wrote the manuscript.
